# Preventive effects of bovine colostrum supplementation in TNBS-induced colitis in mice

**DOI:** 10.1371/journal.pone.0202929

**Published:** 2018-08-23

**Authors:** Iulia Elena Filipescu, Leonardo Leonardi, Laura Menchetti, Gabriella Guelfi, Giovanna Traina, Patrizia Casagrande-Proietti, Federica Piro, Alda Quattrone, Olimpia Barbato, Gabriele Brecchia

**Affiliations:** 1 Department of Veterinary Medicine, University of Perugia, Perugia, Italy; 2 Department of Pharmaceutical Sciences, University of Perugia, Perugia, Italy; 3 Department of Chemistry, Biology and Biotechnology, University of Perugia, Perugia, Italy; University of New Mexico, UNITED STATES

## Abstract

Inflammatory bowel disease (IBD) is a chronic inflammatory disorder for which the current medical therapy is not completely effective. Bovine colostrum (BC) is a biological fluid rich in bioactive molecules that may have beneficial effects on several gastrointestinal disorders. The objectives of this study were to assess the preventive effects of BC supplementation in a mouse model of 2,4,6 trinitrobenzene sulfonic acid (TNBS)-induced colitis using a multidisciplinary approach. Specifically, the following parameters were evaluated: (i) disease activity index (DAI), (ii) histological score, (iii) expression levels of TLR4, anti- and pro-inflammatory cytokines, and (iv) count of some bacterial species of the intestinal microbiota. Mice received a daily suspension of BC (BC group, n = 12) or saline solution (control, CN group, n = 12) for 21 days before the intrarectal inoculation with 1% of TNBS solution. BC was well tolerated and did not induce any histological damage or clinical symptoms. After TNBS treatment, BC group showed a reduction of body weight (BW) loss (P<0.01) and histological score (P<0.05) compared to CN. Moreover, the expression levels of TLR4 (P<0.05), IL-1β (P<0.001), IL-8 (P<0.001), and IL-10 (P<0.001) were lower in mice administered with BC, while the concentrations of TNF-α did not show any differences between groups. Finally, the supplementation with BC resulted in a differential response to TNBS treatment in the bacterial count. In CN group, *E*. *coli* and Enterococci increased (P<0.001), while Anaerobes (P<0.01), Lactobacilli, and Bifidobacteria (P<0.001) reduced. Conversely, no significant changes in bacterial load were found after the inoculation of TNBS in BC pre-treated mice. This study confirms that TNBS-induced colitis model in mice is useful for studying the mechanisms involved in IBD pathogenesis and shows that pre-treatment with BC reduces the intestinal damages and clinical signs of the colitis. Molecular mechanisms and intestinal microflora could be involved in the protective effect of colostrum.

## Introduction

Inflammatory bowel diseases (IBD) are a group of chronic inflammatory idiopathic recurrent disorders of the gastrointestinal tract affecting people worldwide [1]. In humans, Crohn’s disease (CD) and Ulcerative Colitis (UC) are considered the most dangerous conditions included in IBD [2]. Although the exact mechanisms that induce the intestinal inflammation are widely unknown, it is suggested that genetic, environmental, and immunological factors may play a role in the onset of the disease [3–5].

In physiological conditions, the gastrointestinal tract is colonized by commensal microflora that confer important host benefit including mucosal barrier, metabolic and immune regulatory functions, and it may also be potentially exposed to pathogen organisms [6]. As a consequence, the digestive system should be able to downregulate inflammatory responses to commensal bacteria while maintaining the capacity to respond to pathogens in order to preserve the immunological homeostasis [7–9].

Recently, a number of studies suggested that changes of the commensal flora can induce a dysregulation of the intestinal immune response. The shift in the makeup of the intestinal microbiota to a nonphysiologic composition (dysbiosis) could be involved in the pathogenesis of IBD [10]. Indeed, colitis can not be induced in germ-free animals [11], and dysbiosis has been observed in patients affected by IBD [7,12,13].

Microbial agents are recognized by the host immune system through pattern recognition receptors (PRRs). Toll-like receptors (TLRs) are members of PRRs family expressed in both the immune and non-immune cells of different tissues, including those of the gastrointestinal tract [14,15]. TLRs, which are type 1 transmembrane receptors, recognize pathogen-associated molecular patterns (PAMPs) or more extensively microbe-associated molecular patterns (MAMPs) such as carbohydrates, lipids, proteins, and nucleic acids derived from bacteria, viruses, and fungi [16]. To date, various TLRs have been identified in the gastrointestinal tract; however, TLR2, TLR5, and especially TLR4 are considered to be the most related to the onset of IBD [17,18].

TLR4 have a significant role in the host defence and are involved in several intestinal functions such as the maintenance of barrier integrity, immune homeostasis, differentiation, growth as well as the repair of the intestinal epithelium [8,19]. They are expressed on the cell surface of the intestinal epithelial cells of villi, crypts, and stromal cells [20,21]. TLR4 are required for the recognition of LPS, which is the major component of the outer membrane of the cell wall of Gram-negative bacteria [22]. TLR4 are activated by the interaction with a complex constituted by the LPS, LPS-binding protein, and CD14. The activation of the receptor results in the recruitment of different adaptor proteins, including MyD88, TIRAP, TRIF, and TRAM, leading to a cascade of complex intracellular signal pathways that results in the activation of the nuclear factor kB (NF-kB) [23,24]. This induces the transcription of genes involved in host defence, including those for the inflammatory cytokines such as IL-1β, IL-6, IL-8, and TNF-α, chemokines, adhesion molecules, acute phase proteins, antigen presenting molecules, and other inflammatory mediators such as reactive oxygen species as well as nitric oxide in the effector cells [22,23]. In particular, TNF-α is known to promote inflammation by both inhibiting epithelial repair and increasing apoptosis of the cells, which alters intestinal microbiota and facilitates luminal translocation [25]. In IBD illness, TNF-α is found in high levels in mucosa and serum so anti TNF antibody therapy is indeed proved to be a crucial milestone in its treatment [26].

In addition, the organism possesses anti-inflammatory mechanisms which are meant to modulate the magnitude of the response against pathogens. Specifically regulatory T cells (Tregs) are considered a sub-population of T cells, which play a key role in maintaining homeostasis and peripheral tolerance towards self and non-self antigens, by secreting high levels of IL-10 and TGF-β [25]. As a matter of fact, studies proved that mice deficient in IL-10 spontaneously develop a T-cell-dependent colitis, whereas TGF-β suppresses a T-cell-mediated colitis in animals [27]. Moreover, patients with dysfunctional or lack of Tregs develop a severe intestinal inflammation, while treatment with anti TNF-antibodies restore circulating Tregs to normal levels [28,29].

Under homeostatic conditions, there is a counterbalance between the production of pro and anti-inflammatory cytokine. The malfunction of these mediators of inflammation affect the cellular, vascular, and barrier functions other than the immune response, leading both to a disruption of the homeostatic mechanisms and to the breakdown of tolerance to the colonic microflora [19]. In IBD, this leads to a continuous chain reaction with enhanced permeability followed by luminal antigens translocation and a subsequent pro-inflammatory cytokine production that will determine either the onset of a new chronic process or the ongoing of a previous process [30].

At present, therapy protocols are based on the administration of anti-inflammatory drugs, immune system suppressors, antibiotics and supplements, which altogether are not curative, besides, they present many negative secondary effects [31]. A different medical approach to this disease is required, so novel strategies based on the use of natural products such as dietary supplements and food components have been emerging in recent years [32–34].

Colostrum is the first milk rich in a wide range of antimicrobial peptides, immune-regulating components and growth factors with anti-inflammatory and immuno-modulatory properties [35]. The pivotal functions of colostrum are the following: to provide essential nutritional components, to reinforce natural defences, to modulate intestinal microflora and immune responses, and to promote the growth, maturation, and repair of many tissues [36,37]. Recently, several studies have evaluated the effects of BC administration in the prevention and treatment of various gastrointestinal disorders in different animal species and in humans [38–42]. Nevertheless, studies concerning the clinical use of BC in IBD are still limited and controversial [43,44].

The objectives of this study were to assess the preventive effects of bovine colostrum supplementation on the colon inflammation in a mouse model of TNBS-induced colitis using a multidisciplinary approach and combining in vivo and in vitro measurements. More specifically, the following parameters were evaluated: (i) disease activity index (DAI), (ii) histological features, (iii) expression levels of TLR4 and anti- and pro-inflammatory cytokines such as IL-1β, IL-8, TNF-α, and IL-10, and (iv) count of some bacterial species of the intestinal microbiota.

## Materials and methods

### Animals

CD-1 male mice at 6 weeks of age, weighting 34.9±2.42 g (Harlan Laboratoires S.r.l., Correzzana D’Adda, Milan, Italy) were housed in an ambient with the following controlled parameters: 12 h light/dark cycle, 21±1°C constant temperature, and 55±10% relative humidity. All animals were administered standard laboratory chow and water *ad libitum*. After an acclimatization period of 10 days, mice were included in the experimental trial. All experimental protocols were approved by the Ethical Committee for Animal Experimentation at the University of Perugia, Italy. Animal care was in compliance with Italian regulations (Ministerial Declaration 116/92) as well as with European Economic Community regulations (O.J. of European Commission L 358/1 12/18/1986). All efforts were made to minimize animal distress and to use only the number of animals necessary to produce reliable results. A loss of >20% of BW was considered as the criterion for humane endpoint; no mice reached this criterion.

### Bovine colostrum

We used Nutra Summa Pure Bovine Colostrum Powder® (Phoenix, Arizona, USA), which was skimmed, pasteurized, and freeze-dried to ensure minimum denaturation of Ig and of the other bioactive molecules. The concentrations of IgG were 15–20% while the other ingredients are shown in S1 Table.

### Experiment

S1 Fig illustrates the experimental protocol. CD-1 mice (n = 24) were randomly divided into two groups (n = 12) and received daily by gavage a suspension containing bovine colostrum (100 mg of colostrum dissolved in 0.6 mL of saline solution for each mouse, BC group), or the same volume of saline solution (Control group, CN) for 21 days.

BC suspension was prepared immediately before the administration by reconstituting the BC powder with saline solution and vortex mixing; subsequently, it was inoculated by plastic feeding tubes equipped with a 1 ml syringe (2 biological instruments, Basozzo, Varese, Italy). After 21 days of nutritional treatment, six mice of each group (BC pre-TNBS and CN pre-TNBS) were sacrificed by cervical dislocation in order to evaluate the effects of colostrum administration in healthy animals. The other six animals of each group (BC post-TNBS and CN post-TNBS) were treated with TNBS and sacrificed 3 days later to evaluate preventive effects of BC supplementation on the induced colitis. In particular, BC post-TNBS and CN post-TNBS mice were intrarectally inoculated with 150 μl of 1% TNBS solution. TNBS (Sigma-Aldrich, Milan, Italy) solution was prepared at the moment of use diluting the haptinizing agent in ethanol 50%. Before the intrarectal inoculation of 1% TNBS solution, mice were fasted for 24 h and lightly anesthetized with isoflurane (Merial, Milan Italy). A plastic catheter equipped with a 1 ml syringe (2 biological instruments, Basozzo, Varese, Italy) was lubricated and advanced through the anus for 3 cm before the release of the TNBS. In order to ensure the distribution of the TNBS within the colon, the mice were held in a vertical position with the head downwards for one minute after the injection. Body weight (BW) and health status of animals were monitored daily.

### Determination of disease activity index (DAI)

DAI was determined taking in consideration stool consistency, weight loss, and bleeding as previously described by Murano et al. [45] combining their score on a scale from 0 to 4 (S2 Table). The parameters were recorded daily after TNBS treatment (day 22–24).

### Tissue processing

Mice were euthanized 3 days after the TNBS treatment by cervical dislocation. At autopsy, a macroscopic evaluation of the whole digestive tract was made. The gastrointestinal tract was immediately aseptically removed from anus to oesophagus. The large bowel was macroscopically evaluated and then subdivided into caecum, colon, and rectum. Colon was opened longitudinally and the luminal content was removed, immediately placed into an anaerobic chamber, and homogenized in sterile pre-reduced PBS for the bacteriological assays. Colon was washed with saline solution, and representative samples of tissue were collected and fixed in 10% neutral formalin for the histological procedures.

### Histological analyses and scoring

Samples of colon were paraffin embedded, cut in 4–5 μm sections and stained with hematoxylin and eosin (H&E; Merk KGa, Darmstadt, Germany).

Histological scoring was based on a semi-quantitative score system modified by McCafferty et al. [46]. The following features were graded: extent of destruction of mucosal architecture, presence and degree of cellular infiltration, and extent of muscle thickening. For each feature, a score of 0, 1, 2, and 3 was attributed corresponding to normal, mild, moderate, and extensive damage, respectively. The scores for each feature were summed with a maximum possible score of 12.

### Microbiota analysis

Samples of bowel content were aseptically collected, processed via 10-fold dilution and cultured in triplicate. Chromocult agar and Bile Esculin Azide Agar were used for the count of aerobes (E.coli, Coliforms, and Enterococci). The plates were incubated in aerobiosis at 37° C for 24–48 hours. Brain heart Infusion agar, Mann Rogosa Sharpe agar (MRS), modified MRS agar, (0.3% w/v) sodium propionate, 0.2% (w/v) lithium chloride, 0.05% (w/v) cysteine hydrochloride, and 5% (v/v) defibrinated sheep blood were used for the development of the anaerobes (Lactobacillus spp. and Bifidoacterium spp.). The plates were routinely grown using anaerojars and Anaerogen sachets (Oxoid) at 37°C for 48–72 hours. The number of colonies was counted and expressed as CFU x log/g.

### Detection and quantification of gene expression

After rinsing with RNase-free PBS, tissue samples were frozen at -80°C. RNA was extracted from paraffin sections of about 10 μm using FFPE RNA Purification Kit (Norgen Biotek Corp., Ontario, Canada) according to the manufacturer’s instructions with minor adjustments. In order to avoid genomic DNA contamination, the samples were treated with RNase-Free DNase I Kit (Norgen Biotek Corp) following the manufacturer’s instructions. Qubit RNA assay (Life Technologies) was used to measure the amount of RNA, and then, the samples were stored at -80° C until use. A quantity of 20 ng of total RNA was reverse transcribed with iSCRIPT cDNA (Bio-Rad, Hercules, CA, USA). To check for genomic DNA contamination, controls without reverse transcriptase were included, according to the producer’s guidelines.

In order to obtain a total volume of 20 μl for the PCR assay, 1 μl of PrimeTime qPCR IDT (Integrated DNA Technologies, Coralville, Iowa, USA) 250 nM, 10 μl of ITAQ universal probe (Bio-Rad, Hercules, CA), and 5 μL of water were mixed. The following PrimeTime IDT were used for qPCR: IL10 (Mm.PT.58.13531087) TNFα (Mm.PT.58.29509614), IL1ß (Mm.PT.58.42940223), IL8 (Mm.PT.58.33056956), TLR4 (Mm.PT.58.41780308.g), and ACTB (Mm.PT.58.33540333).

In the 96-well PCR plate, reagents were mixed with 4 μl of diluted cDNA (1:10). Each sample was analysed in triplicate. The qPCR analyses were performed with iCycler iQ (Bio-Rad) following these incubation steps: 95°C for 30 seconds, 45 cycles at 95°C for 15 seconds, and finally 60°C, reading fluorescence signal. To confirm the amplification, the resulting PCR product was analyzed by dissociation curves and visualized in an agarose gel. PCR products were purified and sequenced using a QIAquick PCR Purification Kit (Qiagen Inc., Valencia, CA, USA). The relative expression genes were normalized to actin B reference gene levels.

### Statistical analysis

The data was analysed using the Linear Mixed model in which animals were included as random factor [47]. Changes in BW before and after TNBS treatment were evaluated including the Time as repeated factors and BW at day 1 or day 18 as covariate. These models evaluated the effects of Time (days before or after TNBS treatment), Group (2 levels: CN and BC groups), and the interaction between Group and Time. To analyse influences of TNBS on mRNA expressions and bacterial counts of the two groups, the models evaluated the effects of TNBS (2 levels: pre- and post- TNBS treatment), Group (2 levels: CN and BC groups), and their interaction. Sidak adjustment was used for carrying out multiple comparisons. Diagnostic graphics were used for testing assumptions and outliers. Results were expressed as estimated marginal means ± standard error (SE), while raw data were presented in figures. Bacterial counts were expressed and analysed as log10 CFU per gram of colon samples. Mann-Whitney test was used to assess DAI and histological scores. Values were expressed as median (Mdn) and interquartile range (IQR). Statistical analyses were performed with SPSS Statistics version 23 (IBM, SPSS Inc., Chicago, IL, USA). Statistical significance occurred when P ≤ 0.05.

## Results

### Body Weight (BW) and Disease activity index (DAI)

The BW of all mice increased from day 2 to 21 (34.3±0.1 gr and 35.7±0.1 gr at day 2 and 21, respectively; P<0.001); however, there were no differences between groups (34.9±0.1 gr and 35.0±0.1 gr in CN and BC groups, respectively; P = 0.442). Conversely, after TNBS treatment, the BW of BC group was higher than CN group (32.6±0.5 gr and 35.4±0.5 gr in CN post-TNBS and BC post-TNBS groups, respectively; P = 0.003).

After TNBS treatment, 1 of 6 mice died both within CN and BC group. However, although as a trend, DAI of BC group was lower than CN group (Mdn = 1.7, IQR = 1.3–3.3, and Mdn = 0.3, IQR = 0.3–1.3 in CN and BC groups, respectively; P = 0.095).

### Macroscopic evaluation

At macroscopic examination, colon of BC and CN pre-TNBS mice was normal and did not show lesions; whereas, colon of CN post-TNBS mice was swollen, oedematous, and thickened, with evidence of mucosal haemorrhage compared to the BC post-TNBS group at gross examinations.

### Histology

**Fig 1:**
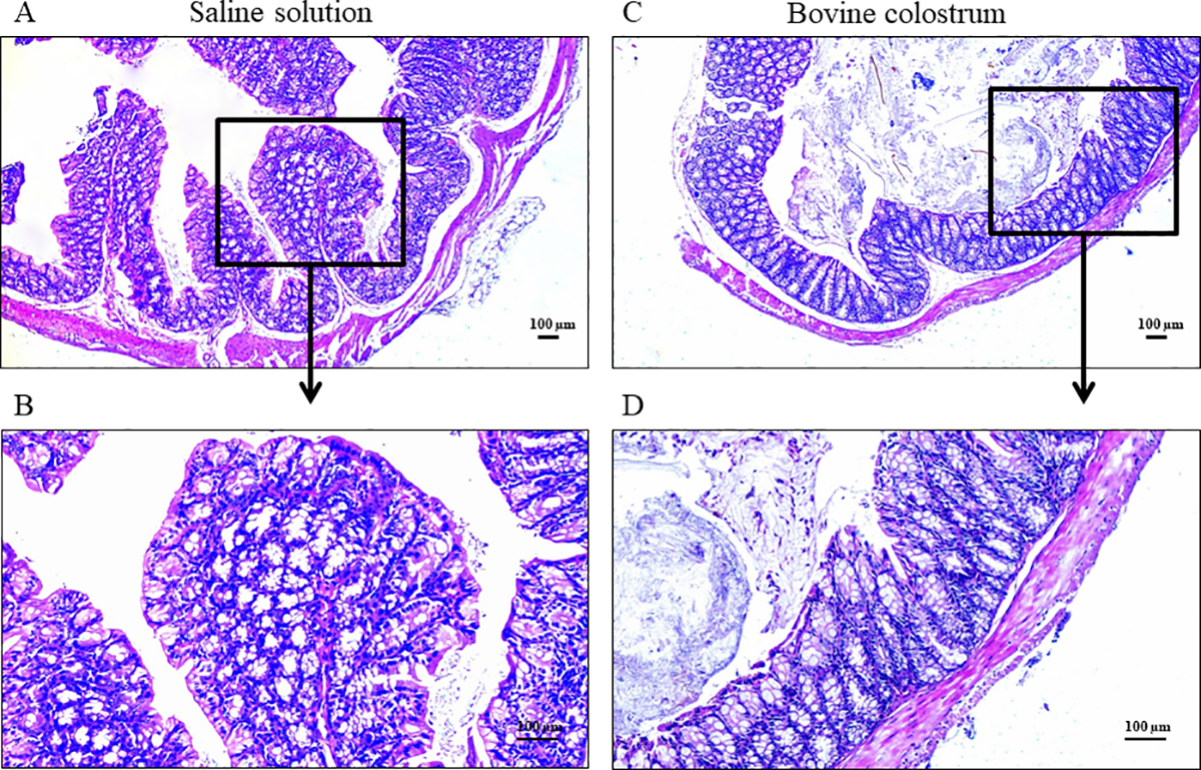
Colon histology before TNBS administration. Comparative assessment of histological aspects from mice treated for 21 days with saline solution (A. General view; B. Detailed aspect; Histological score 2) and Bovine Colostrum (C. General view; D. Detailed aspect; Histological score 1) before the TNBS administration. Both sections revealed a normal structure of the colon with very mild inflammation of the mucosa. H&E stained, magnification of 4x (A-C) and 10x (B-D).

Histological scores of animals sacrificed before TNBS treatment were low in both BC and CN pre-TNBS groups (Mdn = 3, IQR = 2–4, and Mdn = 1, IQR = 0–1 in CN and BC groups, respectively; P > 0.05). The histological examination revealed that the colon of the mice of both groups either appeared normal without structural and morphological alterations or showed very mild signs of inflammation (Fig 1A–1D).

Regardless of the group, histological scores increased after TNBS treatment (Mdn = 1, IQR = 1–3, and Mdn = 4, IQR = 2–4 pre- and post-TNBS, respectively; P = 0.055), while the comparison between groups showed the highest values for CN group (Mdn = 3, IQR = 3–4, and Mdn = 1, IQR = 0–2 in CN and BC groups, respectively; P = 0.024).

**Fig 2:**
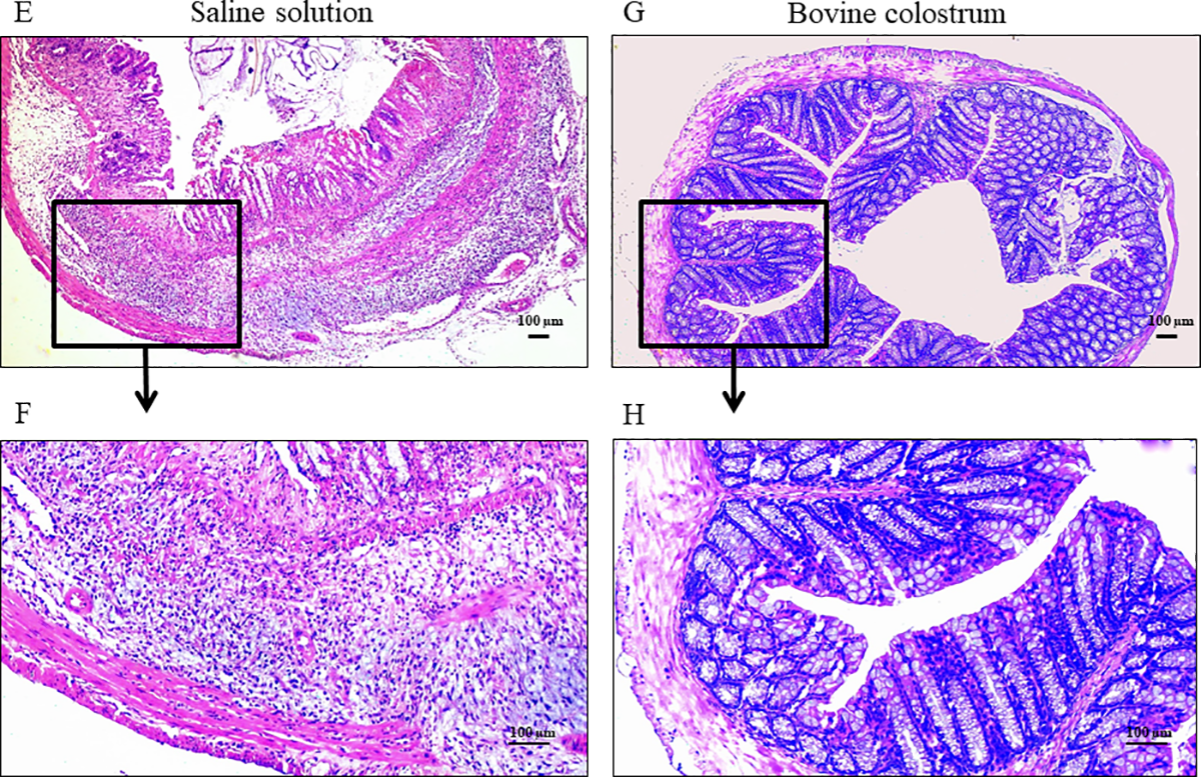
Colon histology after TNBS administration. Comparative assessment of histological aspects of mice treated for 21 days with saline solution (E. General view; F. Detailed aspect; Histological score 4) and Bovine Colostrum (G. General view; H. Detailed aspect; Histological score 3) after the treatment with TNBS. The saline solution group (E-F) showed a severe architectural modification of the mucosa, with focal destruction of the epithelium and crypt loss associated with an inflammatory reaction characterized by severe infiltration of lymphocytes and polymorphonuclear cells (neutrophils and eosinophils granulocytes) in epithelium, lamina propria and submucosal layers. Inflammation also involves partially the muscular layer. Colon of Bovine Colostrum group (G-H) showed evidence of a mild to moderate architectural modification of the mucosa, ulceration of the epithelium, crypt loss accompanied by a mild inflammatory reaction with infiltration of lymphocytes, a few neutrophils and eosinophils granulocytes in lamina propria and submucosa layers. H&E stained, magnification of 4x (E-G) and 10x (F-H).

Histological lesions of the colon of CN post-TNBS mice, resembling IDB, showed a severe grade of inflammation characterized by architectural distortion of the colonic mucosa, erosions of the epithelium, and crypt loss accompanied by a mixed inflammatory infiltrate with lymphocytes and polymorphonuclear cells (neutrophils and eosinophils granulocytes) in the epithelium, lamina propria, and submucosal layer. Inflammation also partially involved the muscular layer (Fig 2E and 2F). On the other hand, colon histology of BC post-TNBS mice showed evidence of a mild to moderate grade of inflammation characterized by infiltration of the mucosa and submucosa by lymphocytes and neutrophilic granulocytes (Fig 2G and 2H).

### TLR4 and cytokines gene expression profiling

**Fig 3:**
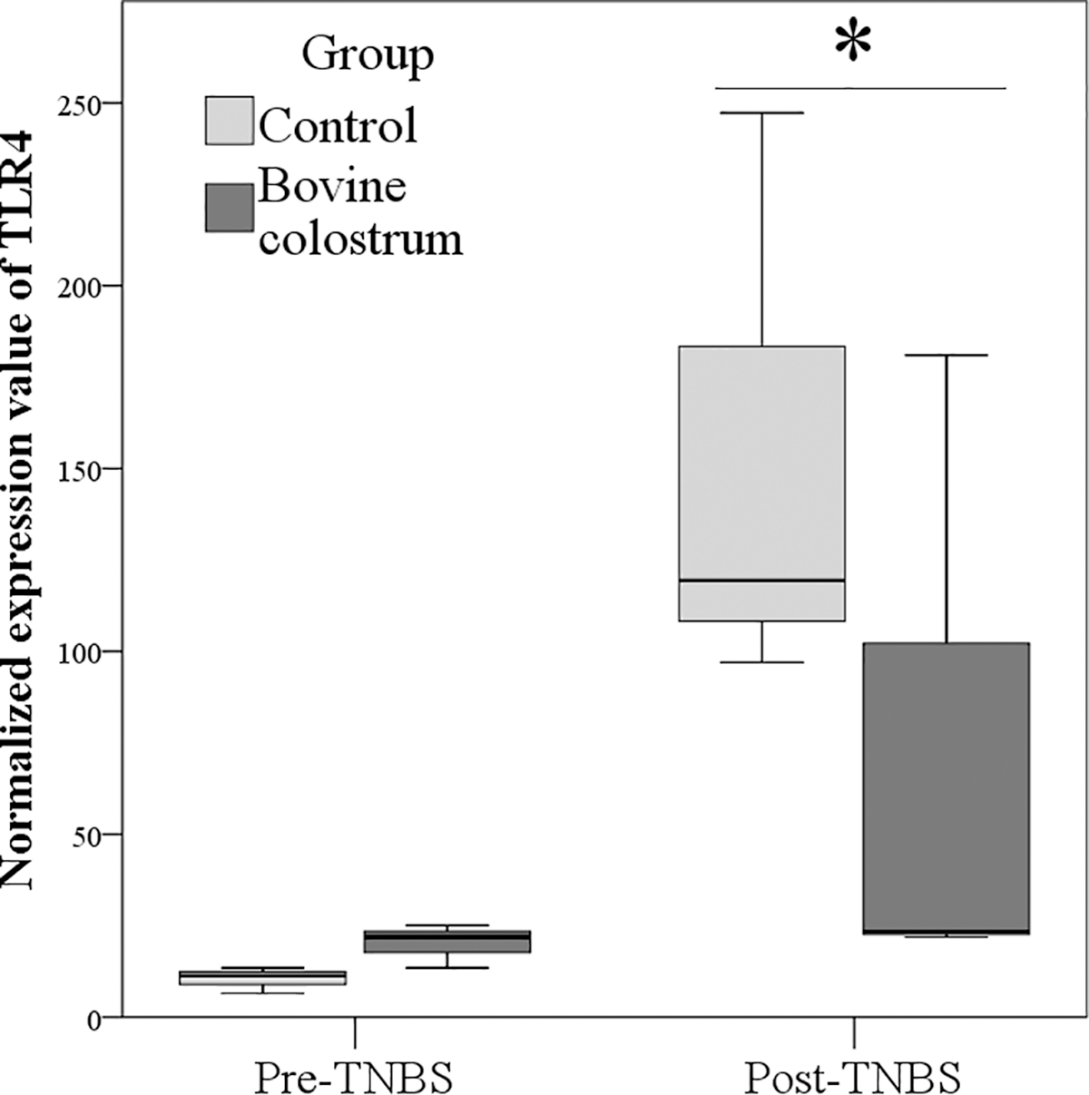
Impact of TNBS treatment on mRNA expression of TLR4 (2^^-ΔCt^) in Control and Bovine Colostrum (BC) groups. *P <0.05. Control *vs* BC group before (Pre) and after (Post) TNBS treatment.

The administration of TNBS increased the expression TLR4 (marginal means: 15.3±20.4 and 115.0±20.4 before and after TNBS, respectively; P<0.01). The normalized value of TLR4 expression differed between groups with lower expression in BC than CN group (P = 0.030; Fig 3). The relative gene expression value was 0.32 (comparative 2^^-ΔΔCt^ method).

**Fig 4:**
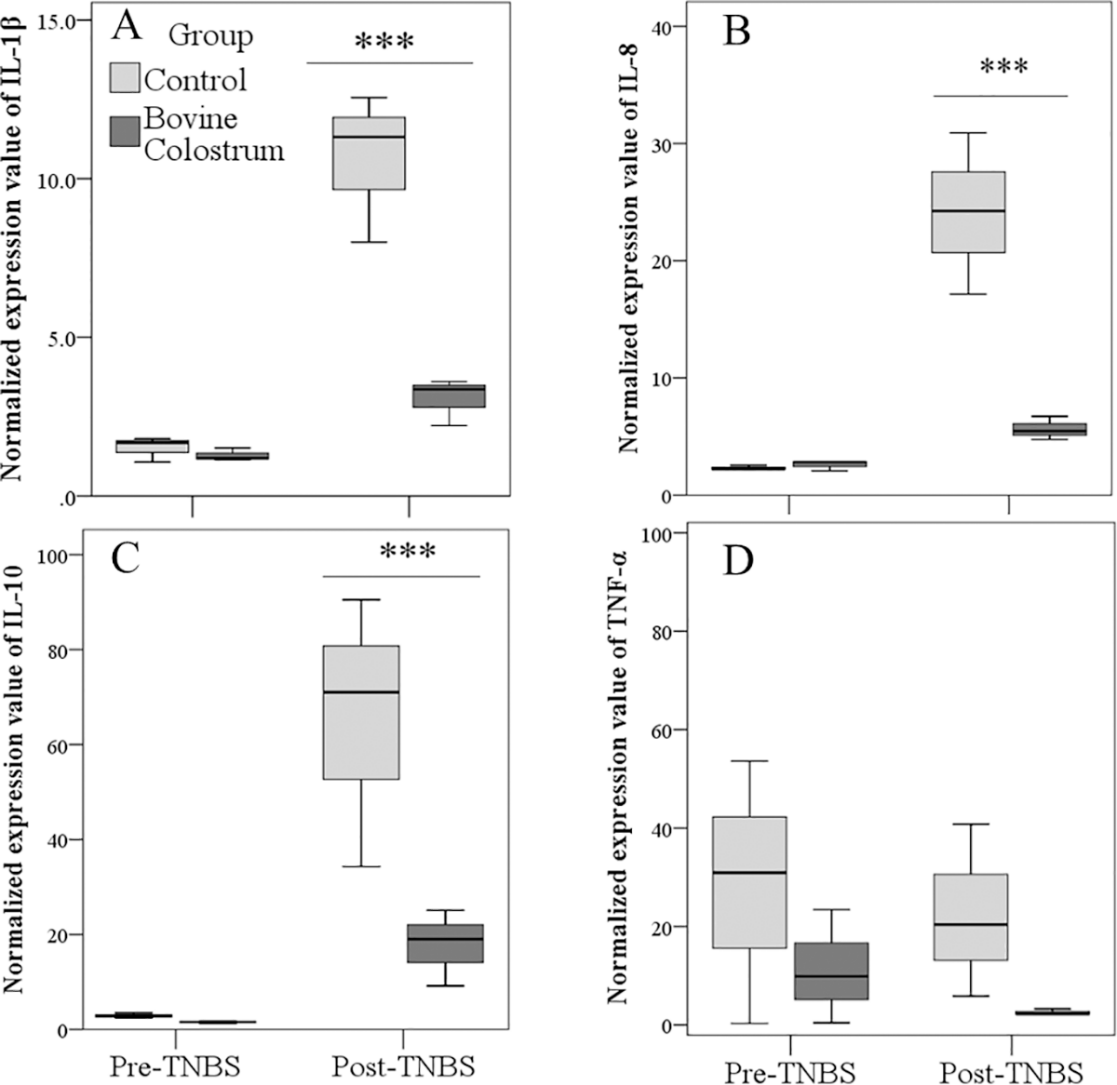
**Impact of TNBS treatment on mRNA expression of IL-1β, IL-8, IL-10, and TNF-α (2^**^**-ΔCt**^**; Panel A-D) in control and Bovine Colostrum (BC) groups.** ***P <0.001. Control *vs* BC group before (Pre) and after (Post) TNBS treatment.

Significant effects of TNBS treatment (P<0.001), Group (P<0.001), and interaction between TNBS and Group (P<0.001) were found for IL-1β, IL-8, and IL-10 mRNA expressions. Marginal means of IL-1β (1.4±0.4 and 6.8±0.4 before and after TNBS, respectively; P<0.001), IL-8 (2.5±1.2 and 14.9±1.2 before and after TNBS, respectively; P<0.001), and IL-10 (2.2±4.9 and 41.5±4.9 before and after TNBS, respectively; P<0.001) were higher after TNBS treatment. Moreover, pairwise comparisons showed higher values of normalized values in CN than BC group after TNBS treatment (P<0.001; Fig 4 Panel A-C). The fold change in the target gene normalized to ACTB and relativized to CN gene expression, calculated for IL-1β, IL-8, and IL-10 sample were respectively 0.29, 0.24, and 0.27 fold change in expression.

A high variability of the expression of TNF-α as well as the effect of neither the group nor treatment were found (Fig 4D).

### Gut microflora

**Fig 5:**
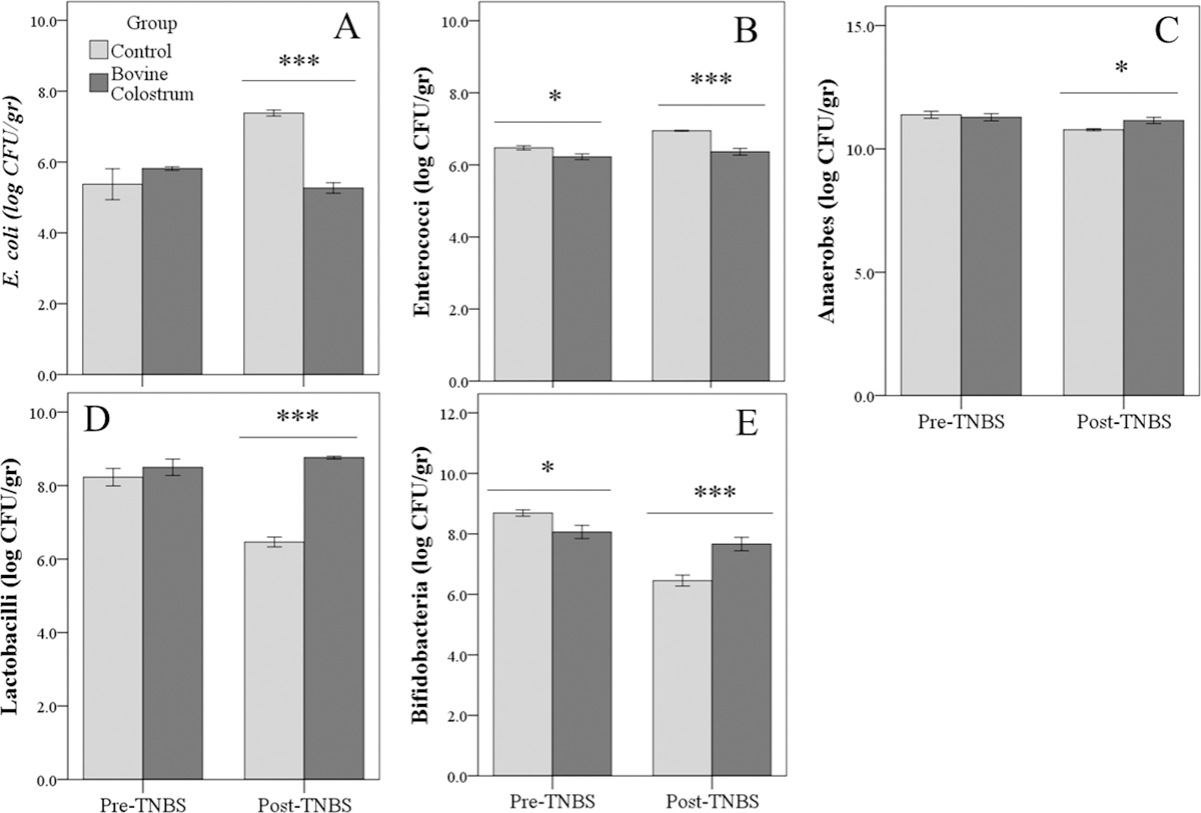
**Impact of TNBS treatment on *E*. *coli*, Enterococci, Anaerobes, Lactobacilli, and Bifidobacteria count (Panel A-E) in control and Bovine Colostrum groups.** Values are mean±SE. *P<0.05, ***P <0.001. Control *vs* Bovine Colostrum group before (Pre) and after (Post) TNBS treatment.

Overall, treatment with TNBS determined an increase in *E*. *coli* (P<0.01) and Enterococci (P<0.001) as well as a reduction in Anaerobes (P<0.01), Lactobacilli (P<0.001), and Bifidobacteria (P<0.001; S3 Table). Group effect showed that estimated marginal means of *E*. *coli* (P<0.001) and Enterococci (P<0.001) were higher in CN than BC group; whereas, Lactobacilli were higher in BC group (P<0.01). Moreover, TNBS resulted in a different response of the mice receiving colostrum for the all the bacteria examined (significant effects for interaction between Group and TNBS treatment: P < 0.05 for Enterococci and Anaerobes; P < 0.001 for *E*. *coli*, Lactobacilli and Bifidobacteria). In particular, after TNBS treatment, no significant changes were found in BC group; conversely, *E*. *coli* and Enterococci increased (P<0.001), while Anaerobes (P = 0.001), Lactobacilli, and Bifidobacteria (P<0.001) decreased in CN group (Fig 5 Panel A-E).

## Discussion

Colostrum contains several biologically active substances such as growth and immunomodulatory factors, Igs, and antimicrobial peptides that retain their activity while passing the gastrointestinal tract carrying out beneficial effect on the intestinal functions [35,36,48]. Colostrum and colostrum derived products have been used for the prevention and treatment of several gastrointestinal disorders [39,41,42,44,49,50]. However, experimental trials for the treatment of IBD are still limited. Thus, in our study, we determined whether the oral administration of BC may prevent the clinical signs in a murine model of TNBS-induced colitis using a multidisciplinary approach.

Our study showed that BC administration was well tolerated and did not induce any damage or pathological symptoms. In particular, we did not observe BW loss, changes in intestinal microbiota, histological score, and DAI before the treatment with TNBS. According to the current state of knowledge, BC appears to be safe and no contraindications are observed even when it is administered in high concentrations both in humans and animals [44,51,52]. Only a few studies have reported lactose intolerance, nausea, flatulence, transient diarrhoea, and unspecified abdominal discomfort as possible side effects [49,53].

The key finding of our study was that the preventive BC administration reduced the clinical signs in TNBS-induced colitis in CD1 mice. In particular, after TNBS treatment, BW loss appeared to be less severe, and both DAI and histological score were reduced in BC-treated mice in comparison to control group. Our results are in agreement with other studies conducted in human IBD and in Dextran Sulphate Sodium (DSS)-induced colitis in mice [43,44]. Similar findings were also found in a murine model of TNBS-induced colitis using hyperimmune BC although they were different in animals administered with raw BC [54].

There is evidence that both immune system and microbiota are involved in the inflammatory process that leads to intestinal lesions in IBD. In fact, dysbiosis and defects in mucosal immune functions such as microbe recognition, barrier function, and antimicrobial effector mechanisms may play a crucial role in the pathogenesis of IBD [5,9]. It is speculated that the chronic inflammation in IBD could be either a normal immune response to an abnormal intestinal microbiota or, in other cases, an excessive and aberrant immune response to a normal commensal flora in genetically susceptible individuals [5,7,9]. LPS from outer membrane of gram-negative bacteria can induce the activation of the innate immune system binding TLR4 in immune and non-immune cells. The activation of TLR4 by LPS leads to the induction of genes that function in the host defense including pro-inflammatory cytokines, chemokines, and other inflammatory mediators that influence the immune response and make a link between innate and acquired immunity [15]. As a consequence, an upregulation of TLR4 expression can be involved in the process of abnormal and enhanced inflammatory responses [18,22,55]. In a murine model of DSS-induced colitis, an increase in mRNA and protein expression in intestinal epithelial cells for TLR4 that was correlated with IL-1β expression was demonstrated [21]. Moreover, an up-regulation of TLR4 and pro-inflammatory cytokines was also found in other studies [56–58]. However, TLR4 responses to LPS of commensal bacteria also play a crucial role in the maintenance of both the intestinal homeostasis and host tolerance [8]. Indeed, gut microbial LPS is considered to be a strong activator of the innate immune system as well as a component of the microbiota which is able to influence the host physiology. Finally, LPS tolerance can be considered one of the mechanisms that may control the development of IBD preventing an abnormal activation of the immune system [59]. For this reason, the down-regulation of TLR4 expression and/or a negative regulation of their intra-cellular signalling pathways and functions could be necessary [60–62].

In this context, another remarkable finding of our study was that the prophylactic administration of BC reduced both the gene expression of TLR4 (0.32 fold change) and of some pro-inflammatory cytokines such as Il-1β (0.29 fold change) and IL-8 (0.24 fold change) after the treatment with TNBS compared to the control group. These results are consistent with previous reports in which BC reduced the protein expression not only of TLR4 but also of different pro-inflammatory cytokines such as L-1β, IL-8, and TNF-α in the small intestine of preterm pigs [42]. Moreover, it appears that BC whey protects the integrity of the intestinal mucosal barrier by inhibiting the expression levels of inflammatory genes following the invasion by enteric bacterial pathogens [63,64]. Colostrum proteins inhibit NF-κB-mediated pro-inflammatory cytokine expression and switch off markers of inflammation [65]. An inhibition of pro-inflammatory cytokines was also reported in a DSS model using Balb mice administered with colostrum whey and other components of milk [66]. Finally, in an *in vitro* study, An et al. [67] showed that colostrum blocks IL-1β-induced pro-inflammatory gene expression and COX-2 protein expression in human colon cancer cell line HT-29 through an inhibition of NF-κB signaling pathway. Similar results were also found in mice [38]. Moreover, BC contains considerable high levels of IgG that may be increased by the vaccination of the cows during pregnancy with the aim to enhance the concentrations of specific antibodies [68,69]. The central role of Igs in the intestine is to bind the bacteria, in particular the pathogens, with a mechanism called exclusion. In this way, the bacteria can not enter into contact with the intestinal epithelium and it is reduced the continuous antigenic stimulation from the gut-derived bacterial endotoxin such as LPS, favouring the maintenance of a state of tolerance [70]. Moreover, Igs can maintain the intestinal barrier functions reducing the risk of inflammation and bacterial translocation [71]. IL-10 is an anti-inflammatory cytokine that plays a crucial role in the intestinal immune homeostasis. Indeed, IL-10 deficiency leads to the development of colitis in mice [72]. The inhibitory cytokines IL-10, TGFβ and IL-35 are considered to be Tregs pivotal mechanism of annihilating pro-inflammatory cytokines secretion of other immune cell types [73,74]. Our results showed an increase of IL-10 expression after TNBS treatment both in control and BC groups of mice although the control group showed a higher value. In a previous study, hyperimmune BC, although not raw colostrum, promoted the increase of Tregs and the serum levels of IL-10 in mice [54]. On the other hand, previous data did not reveal a significant increase of IL-10 in mice prophylactically treated with BC [75].

In the host, intestinal microbiota play crucial roles not only in inhibiting colonization by several pathogens but also in conferring mucosal barrier as well as metabolic and immune regulatory functions [6,8,10]. It is believed that specific bacteria in the gut microbiota such as Lactobacilli and Bifidobacteria can regulate and direct both the barrier functions and the activity of the immune system contributing to the maintenance of the intestinal homeostasis [38]. For these reasons, the manipulation of the intestinal flora favouring the development of specific bacterial strains that can impact immune function counteracting the dysbiosis and the resulting inflammation can be an attractive strategy of clinical interest [10,76]. BC contains abundant concentrations of oligosaccharides such as fructo-oligosaccharides and beta-galacto-oligosaccharides [77], gangliosides [78], and nucleosides [79] which have been claimed to benefit the health of the colon by selectively stimulating the growth of beneficial bacteria acting as prebiotics. In our study, the administration of TNBS in the control group created an imbalance of intestinal microbiota, which is similar to that observed in IBD, consisting in the increase of *E*. *coli* population and in the decrease of Lactobacilli and Bifidobacteria. In fact, similar results were found in IBD patients and in TNBS induced colitis in mice [76] which presented a reduction in the biodiversity and a depletion of both some bacterial phyla in feces and mucosa-associated microbiota compared to healthy individuals [12,13]. On the other hand, BC administration seems to preserve the intestinal microbiota, especially the beneficial bacteria. We can, thus, hypothesize that BC can stabilize intestinal microbiota favouring the growth of beneficial bacteria which in turn may contribute to reinforce the mucosal barrier function and to modulate the immune response reducing the severity of the inflammatory reaction. As previously mentioned, the beneficial gram-negative bacteria increases the nonimmunogenic LPS concentration that may reduce the cell surface expression level of the LPS receptor complex and therefore the activation of the TLR4 signalling pathway. This downregulation contributes to the maintenance of the immunological tolerance state. Indeed, a recent study showed that a proinflammatory cellular pathway that is usually activated by pathogenic bacteria is actively repressed by some bacteria species of the intestinal microbiota; this contributes to the idea that beneficial microbes play a role in the establishment of immune tolerance and homeostasis in the gut [80]. Moreover, some gram-negative bacteria are able to produce antagonistic forms of LPS which can inhibit TLR4 signaling while some commensal bacteria and pathogens are able to produce LPS variants that prevent the inflammatory endocytosis of TLR4 mediated by CD14 [81,82].

Our results are in accordance with recent studies which provided evidence that BC administration might balance the indigenous microbiota [64,83–85]; however, other studies reported no detectable difference in gut bacterial composition in piglets treated with hyperimmune BC [86].

## Conclusion

Bovine colostrum is a rich source of biologically active molecules that are essential for several specific functions of digestive system and, at the same time, that can synergistically intervene in various pathogenetic phases of the IBD. BC may has the potential to modulate the immunological response as well as the severity of the intestinal inflammatory reaction modulating TLR4 and cytokine expression, reducing BW loss and histological score, balancing the microbiota, and finally decreasing the clinical signs of colitis in mice. Moreover, BC seems a suitable candidate to be used as a natural supplement that may reduce the side effects of the synthetic drugs that are currently used in the treatment of IBD and other digestive system diseases although its effectiveness and safety still need to be demonstrated.

Despite the encouraging results, further studies are required not only to confirm the effectiveness and safety of BC but also to clarify the immunomodulatory mechanisms that can prevent the development and the progression of the chronic inflammation in IBD.

## Supporting information

S1 Fig Experimental Protocol. After an acclimatization period of 10 days, 24 CD-1 male mice were randomly divided into two groups (n = 12) and daily received by gavage a suspension containing bovine colostrum (BC group) or the same volume of saline solution (CN group) for 21 days. After 21 days, 6 mice of each group (BC pre-TNBS and CN pre-TNBS) were sacrificed. The other 6 animals of each group (BC post-TNBS and CN post-TNBS) were treated with TNBS and sacrificed 3 days later.
(TIF)

S1 Table The concentrations of Ig and other major ingredients of BC. (PDF)

S2 Table Disease activity index. Parameters and criteria for scoring Disease activity index.
(PDF)

S3 Table Estimated marginal means ± standard error (SE) and P-values for TNBS (2 levels: pre- and post- TNBS treatment) and Group (2 levels: Control and BC groups) effects of bacterial counts (log10 CFU per gram of colon samples). (PDF)
