# Supplementary figures and images for: Preventive effects of bovine colostrum supplementation in TNBS-induced colitis in mice
Source: PLoS One. 2018 Aug 23;13(8):e0202929. doi: 10.1371/journal.pone.0202929 (PMC6107273; doi:10.1371/journal.pone.0202929)

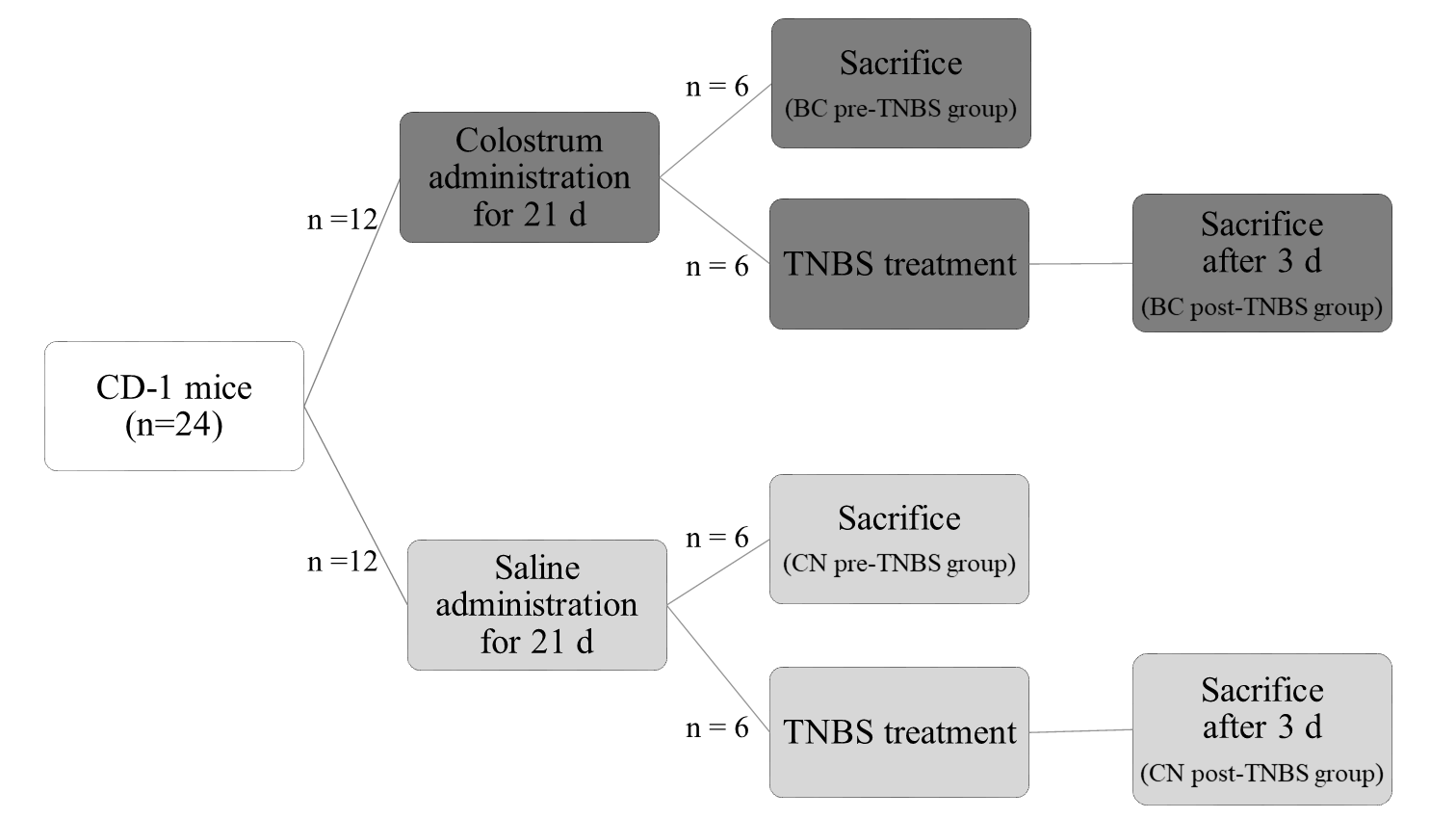

Supplement: S1 Fig — After an acclimatization period of 10 days, 24 CD-1 male mice were randomly divided into two groups (n = 12) and daily received by gavage a suspension containing bovine colostrum (BC group) or the same volume of saline solution (CN group) for 21 days. After 21 days, 6 mice of each group (BC pre-TNBS and CN pre-TNBS) were sacrificed. The other 6 animals of each group (BC post-TNBS and CN post-TNBS) were treated with TNBS and sacrificed 3 days later. (TIF) [file pone.0202929.s001.tif]
