# Supplementary material for: Preventive effects of bovine colostrum supplementation in TNBS-induced colitis in mice
Source: PLoS One. 2018 Aug 23;13(8):e0202929. doi: 10.1371/journal.pone.0202929 (PMC6107273; doi:10.1371/journal.pone.0202929)
Supplement: S1 Table — (PDF) [file pone.0202929.s002.pdf]

S1 Table. The concentrations of Ig and other major ingredients of BC.

Components	Per 100 g
**Energy**	500 kcal
	2092 kJ
**Protein**	46 g
**Total Fat**	24 g
**Carbohydrates**	20 g
**Sugars**	20 g
**Sodium**	400 mg
**IgG**	15-20 %
