# Supplementary material for: Preventive effects of bovine colostrum supplementation in TNBS-induced colitis in mice
Source: PLoS One. 2018 Aug 23;13(8):e0202929. doi: 10.1371/journal.pone.0202929 (PMC6107273; doi:10.1371/journal.pone.0202929)
Supplement: S2 Table — Parameters and criteria for scoring Disease activity index. (PDF) [file pone.0202929.s003.pdf]

S2 Table. Disease activity index. Parameters and criteria for scoring Disease activity index.

PARAMETER	CRITERIA	SCORE
**Weight loss (%)**	<1	0
	1-5	1
	6-10	2
	11-15	3
	>15	4
**Stool consistency**	Well-formed pellets - Diarrhoea	0-4
**Rectal bleeding**	Negative - Gross bleeding	0-4
**DISEASE ACTIVITY INDEX (DAI)**	(Score of Weight loss + score of Stool consistency + score of Rectal bleeding) / 3	0-4

Modified by Murano et al. (2000)
