# Supplementary material for: Preventive effects of bovine colostrum supplementation in TNBS-induced colitis in mice
Source: PLoS One. 2018 Aug 23;13(8):e0202929. doi: 10.1371/journal.pone.0202929 (PMC6107273; doi:10.1371/journal.pone.0202929)
Supplement: S3 Table — (PDF) [file pone.0202929.s004.pdf]

S3 Table. Estimated marginal means ± standard error (SE) and P-values for TNBS (2 levels: pre- and post- TNBS treatment) and Group (2 levels: Control and BC groups) effects of bacterial counts (log10 CFU per gram of colon samples).

**Bacteria**	**Factors**					
	**TNBS treatment**			**Group**		
	**Pre-TNBS**	**Post-TNBS**	**P value**	**Control**	**Bovine colostrum**	**P value**
E. coli	5.6±0.1	6.3±0.1	0.001	6.4±0.1	6.3±0.1	<0.001
**Enterococci**	6.4±0.1	6.7±0.1	<0.001	6.7±0.1	6.3±0.1	<0.001
**Anaerobes**	11.3±0.1	11.0±0.1	0.002	11.1±0.1	11.2±0.1	0.196
**Lactobacilli**	8.4±0.1	7.6±0.1	<0.001	7.3±0.1	8.6±0.1	<0.001
**Bifidobacteria**	8.4±0.1	7.1±0.1	<0.001	7.6±0.1	7.9±0.1	0.106
